# Intrathecal methotrexate injection combined treatment of gastric cancer with leptomeningeal metastasis: case report

**DOI:** 10.3389/fonc.2024.1464376

**Published:** 2024-12-19

**Authors:** Wenting Xie, Xun Kang, Sijie Huang, Wenbin Li

**Affiliations:** Department of Neuro−Oncology, Cancer Center, Beijing Tiantan Hospital, Capital Medical University, Beijing, China

**Keywords:** leptomeningeal metastasis (LM), gastric cancer, intrathecal methotrexate injection, brain tumor, case series

## Abstract

**Background:**

Leptomeningeal metastasis of gastric adenocarcinoma (LM-GC) is a rare and severe complication with a poor prognosis, its prognosis is significantly poorer than liver, lung, and peritoneal metastases. Studies on LM-GC have been limited to clinical case reports. Despite advances in systemic therapies, there is a lack of standardized treatment protocols for LM-GC due to its rarity and the challenges it presents.

**Methods:**

This case series reports on six patients diagnosed with LM-GC who received intrathecal methotrexate (MTX) injection from June 2018 to November 2023. Treatment efficacy, safety, and prognostic factors were analyzed by comparing symptoms and laboratory test results before and after treatment.

**Results:**

The average OS for the cohort was 7.5 months, exceeding previous reports for LM-GC patients, which has a median survival time of 4-6 weeks. No significant changes were observed in cerebrospinal fluid glucose and chloride levels post-MTX treatment. However, a statistically significant decrease in cerebrospinal fluid protein levels was noted after treatment (P < 0.05). Adverse reactions were mild, with the most common being bone marrow suppression and oral mucosal ulcers.

**Conclusion:**

Intrathecal MTX injection combined treatment offers a potential therapeutic strategy for LM-GC, improving clinical symptoms and extending survival. Further research is warranted to validate these findings and explore the molecular mechanisms of LM-GC for targeted therapies.

## Introduction

Gastric cancer remains to be one of the primary cancer-related mortality worldwide, with its aggressive nature and high metastatic potential. As the fifth most common malignant neoplasm globally, there emerge approximately 970,000 new cases and 660,000 deaths of gastric cancer annually (1). By 2040, it is anticipated that there will be a staggering 7.5 million new cases of gastrointestinal tract cancers worldwide, resulting in an estimated 5.6 million deaths (2). Despite advances in diagnostic and therapeutic modalities, the prognosis for patients with advanced gastric cancer remains poor, particularly with the central nerve system involved. Although the frequency of leptomeningeal metastasis in gastric cancer (LM-GC) is only 0.16-0.069% (2, 3), patients with leptomeningeal metastasis have a median survival time of 4-6 weeks (4). However, due to the low incidence and poor prognosis of leptomeningeal metastasis from gastric cancer, there are very few studies on this topic internationally. Reports on gastric cancer metastasis have been limited to clinical case reports. The rarity of LM-GC poses challenges in establishing standardized treatment protocols, and as such, the management of these patients often relies on case-specific decisions.

Therefore, intrathecal injection therapy has garnered significant attention, which involves the direct injection of medication into the subarachnoid space, can increase the drug concentration in the cerebrospinal fluid while minimizing systemic toxicity (5). The introduction of intrathecal chemotherapy, specifically methotrexate (MTX), has shown promise in the treatment of LM from various primary malignancies (3, 5). However, due to the lack of prospective clinical trials and high-level evidence, many treatment recommendations are primarily based on expert opinions and consensus (3, 6). Moreover, because patients with LM often have extensive multi-organ metastases and a short survival period, analyzing the efficacy of intrathecal drug therapy for LM is challenging. Therefore, our study retrospectively analyzes the clinical efficacy of intrathecal injection therapy for gastric cancer patients in our center, aiming to provide a basis for further clinical treatments.

## Subjects and methods

A total of 6 patients with LM-GC were included in the study. All patients had received MTX for intrathecal injection (IT). The options for the combination treatment included oxaliplatin, capecitabine, S-1, paclitaxel and ACB treatment. We administered intraventricular injections of methotrexate 10mg plus dexamethasone 5mg in all the patients who met the following criteria: 1) no bleeding tendency; 2) no rapid progression of the lesions other than LM.

All patients were followed up either at the outpatient clinic or by telephone communication. The follow−up period of all 6 patients was concluded on November 24, 2023. All 6 patients and their kin gave their consent for the utilization of their medical data for research. We employed an intrathecal chemotherapy regimen that includes the administration of methotrexate 10mg plus dexamethasone 5mg via intrathecal injection. The treatment is structured in phases:Induction Phase (First Month): The intrathecal chemotherapy is administered twice a week. Consolidation Phase (Second Month): The frequency is reduced to once a week. Maintenance Phase (Thereafter): The treatment frequency is further reduced to once a month. In the event of disease progression, the intrathecal chemotherapy can be restarted from the induction phase and continued until the patient no longer visits our center for treatment. Overall survival (OS) was defined as the duration between the LM−GC diagnosis and death. Before and after the intrathecal chemotherapy, cerebrospinal fluid (CSF) biochemical indicators such as protein, glucose, and chloride levels were recorded. Adverse reactions were evaluated using the Common Terminology Criteria for AEs (CTCAE, version 5.0). Overall survival was calculated from the date of diagnosis of the LM-GC by CSF cytology or MRI to the date of death. The median overall survival was calculated by the Kaplan–Meier method, using SPSS version 25.0 (IBM Corp, Armonk, NY). All statistics were two−sided, and P < 0.05 was considered statistically significant.

## Clinical features, treatments, and outcomes of 6 patients

Four males and two females with a median age of 51 years (range, 38–70 years) were included in our study (**Table 1**). Four patients had poor differentiated adenocarcinoma, one had moderate to poor differentiated adenocarcinoma, and one patient had moderate differentiated adenocarcinoma. At the time of diagnosis of the LM-GC, other metastatic disease was also observed in all 6 patients, including lung (n = 1), lymph node (n = 1), cutaneous (n = 1), bone (n = 3), peritoneal (n=3) and liver metastasis (n = 2).

**Table 1:** Summary of 6 patients of leptomeningeal metastasis from Gastric adenocarcinoma.

No.		1	2	3	4	5	6
Sex/age (yr)		female/72	male/57	male/48	male/55	female/38	male/50
Interval from Gastric adenocarcinoma to LM dx (mo)		24	5	29	27	2	3
Clinical presentations		Decreased vision, Epilepsy, Headache, Dizziness, Vomiting, Dysphagia, Disturbance of consciousness	Headache, Impaired fluency of speech, decreased calculation ability and memory.	Fatigue, Diplopia, Strabismus	Headache, Vomiting, Hearing Loss	Nausea, Vomiting, Decreased vision	Dizziness, Headache, Urinary Incontinence, Decreased vision
Karnofsky Performance Score		50	70	60	50	40	80
Brain parenchyma involvement		None	None	Left Cerebellum, Right Frontal Lobe	None	Right Temporal Lobe	Bilateral Occipital Lobes
Extra-CNS metastases		Cervical Vertebral Body Metastasis	Lung, Bone, Peritoneal, Liver Metastasis	Bone Metastasis	Peritoneal, Liver Metastasis	Peritoneal, Lymph nodes Metastasis	Cutaneous Metastasis
Primary pathology		Moderate Differentiated Adenocarcinoma	Poor Differentiated Adenocarcinoma	Moderate to Poor Differentiated Adenocarcinoma	Poor Differentiated Adenocarcinoma	Poor Differentiated Adenocarcinoma	Poor Differentiated Adenocarcinoma
Treatment prior to the diagnosis of the LM		None	Chemotherapy	Chemotherapy, Immunotherapy	Chemotherapy, Immunotherapy	None	Chemotherapy
Treatment after the diagnosis of the LM		Intraventricular injections of methotrexate	Chemotherapy, intraventricular injections of methotrexate	Chemotherapy, Immunotherapy, intraventricular injections of methotrexate	Chemotherapy, Immunotherapy, intraventricular injections of methotrexate	Chemotherapy, Immunotherapy, intraventricular injections of methotrexate	Chemotherapy, Immunotherapy, intraventricular injections of methotrexate
Ommaya reservoir		yes	yes	yes	yes	none	yes
Doses of IT received (count)		3	13	4	7	9	16
Time from LM dx to endpoints (mo)		7	13	8	3	4	10
Tumor Biomarker	CEA ng/mL	3.42	7.03	4.09	47.51	6.75	6.52
	CA12-5 U/mL	13.72	27.72	6.92	9.79	176.1	14.29
	CA19-9 U/mL	5645	14.86	7.9	424.8	7.55	14057
	CA72-4 U/mL	40.88	2.06	9.39	1927	4.9	7.9

**Figure 1:**
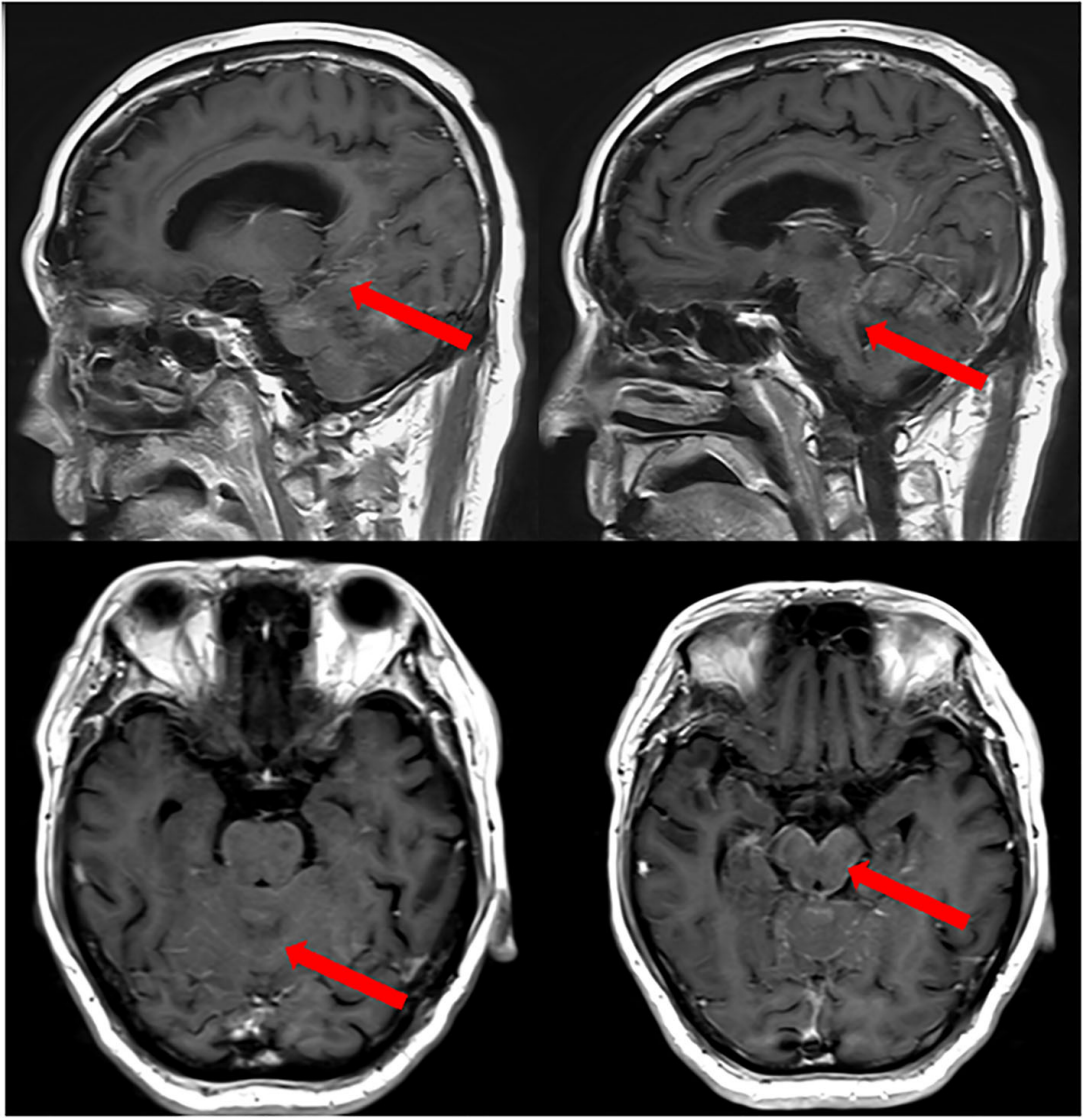
MRI of Case 4 patient with LM−GA. An obvious line−like enhancement was observed on the surface of the cerebellum and pons before the IT combination treatment (red arrows).

Headache was noticed in 4 patients, physical disability in 4, cranial nerve involvement in 4, vomiting in 3 patients. Other patients also exhibit a variety of neurological symptoms such as epilepsy, deafness, diplopia, incontinence of stool and urine, and loss of consciousness, depending on the extent of the affected nervous system. Three patients had a KPS score of <60, indicating poor physical functioning. Brain parenchymal metastasis was observed in 3 of patients. Furthermore, an intraventricular Ommaya reservoir was implanted in 5 patients. Initial LM-GC diagnosis was made by enhanced MRI in all six patients, the MRI of case 4 patient was shown in **Figure 1** as an example. Among all the patients diagnosed with leptomeningeal metastasis, tumor markers were abnormal in six cases, with four cases showing elevated CA72-4, three cases with elevated CA19-9, five cases with elevated CEA, and one case with elevated CA12-5.

**Figure 2:**
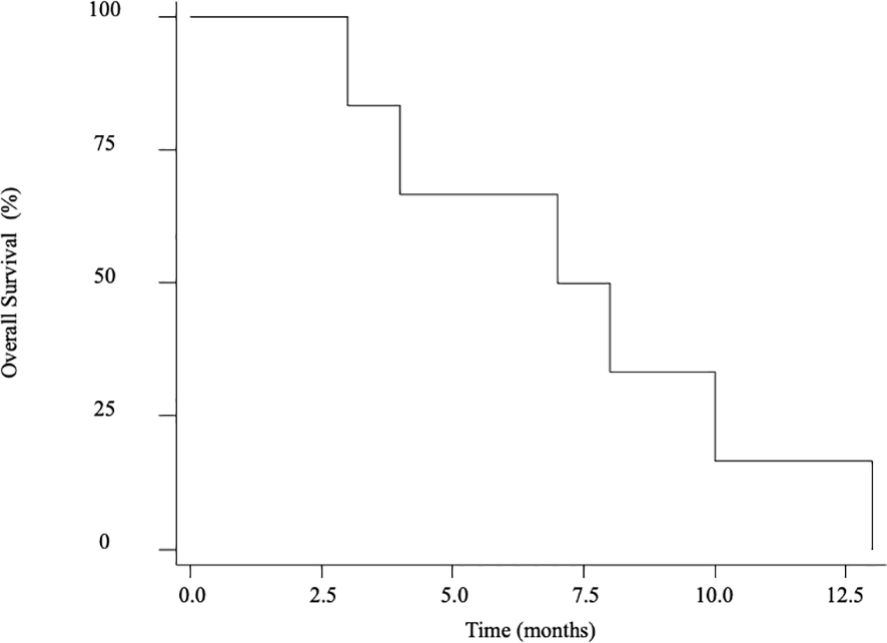
Overall survival for the entire cohort (n = 6).

**Figure 3:**
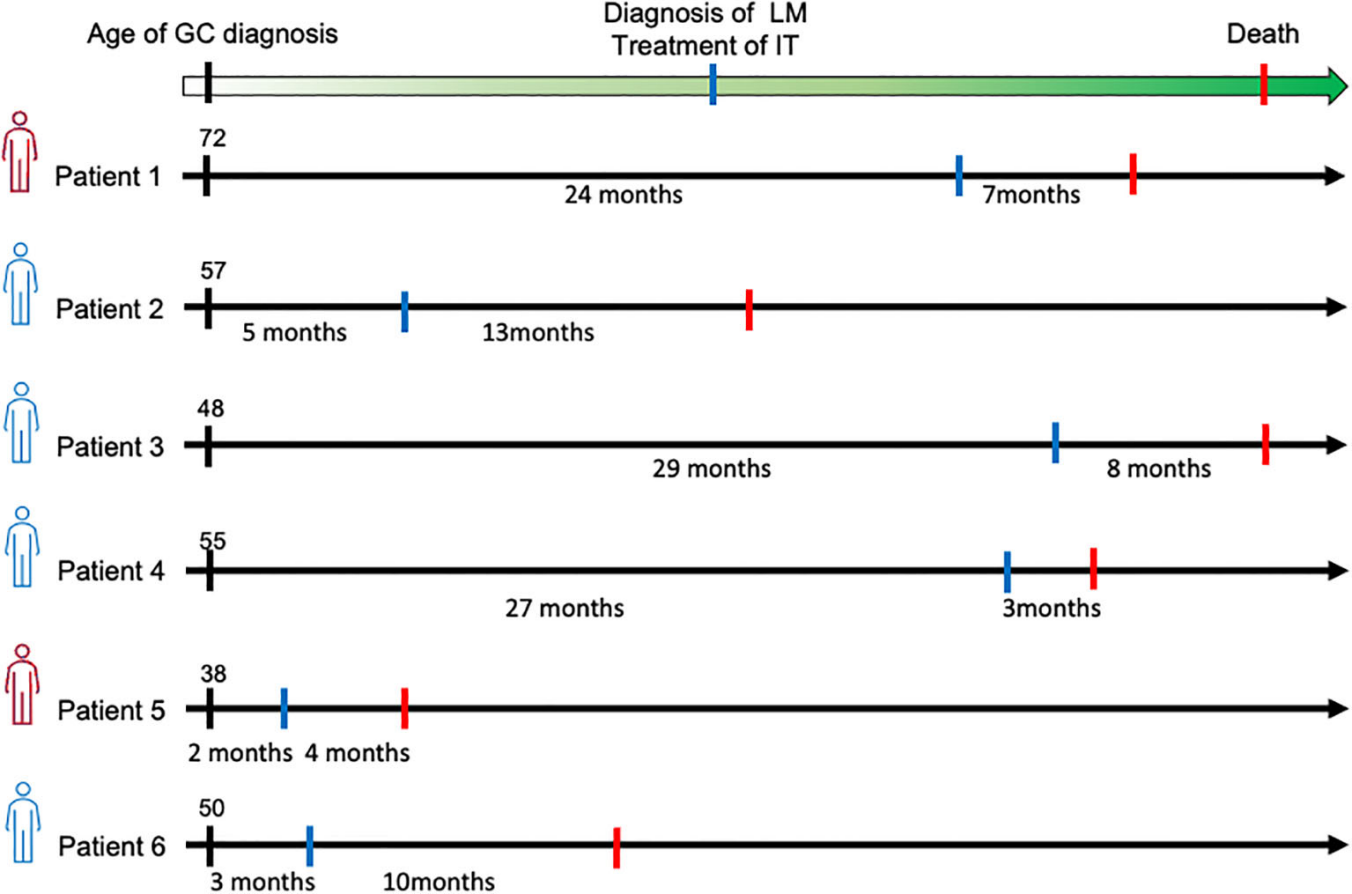
Survival schema of six patients.

Of the 6 patients, 2 had received chemotherapy and 2 had chemotherapy combined with immunotherapy for gastric cancer prior to the diagnosis of the LM. At the onset of LM, the efficacy of the previous therapy was rated as partial response or stable in 3 patients, progress in one patient. The other 2 patients who did not receive any previous therapy were detected with leptomeningeal metastasis shortly after the gastric cancer diagnosis. The median interval from gastric cancer to LM-GC was 14.5 months (range, 2–29 months). The therapeutic modalities applied for the 6 patients were as follows: IT alone in 1 patient, IT plus Chemotherapy in 1 patient, and IT plus Chemotherapy plus Immunotherapy in other 4 patients. The median dose of IT is 8 counts (range, 3-16 counts). The average overall survival for the entire cohort (n = 6) is 7.5 months (**Figure 2**). And the survival schema of six patients were shown in **Figure 3**.

Before and after MTX treatment, no changes were found in the glucose and chloride levels in the cerebrospinal fluid of the enrolled patients. The results of the cerebrospinal fluid biochemical tests showed that before the intrathecal injection of MTX, the average cerebrospinal fluid protein was 114.82 mg/L, and the average lowest level of protein in the cerebrospinal fluid after treatment was 41.172 mg/L, the difference was statistically significant (P < 0.05, **Table 2**).

**Table 2:** Levels of cerebrospinal fluid biochemical markers before and after intrathecal methotrexate injection therapy.

Characteristics	Before IT	After IT	P value
n	6	6	
CSF-Pro (mg/L), mean ± sd	114.82 ± 74.434	41.172 ± 16.587	0.040
CSF-Glu (mmol/L), mean ± sd	3.1433 ± 1.1149	3.9133 ± 1.3153	0.300
CSF-Cl (mmol/L), mean ± sd	118.33 ± 4.8442	122.83 ± 5.4924	0.163

**Figure 4:**
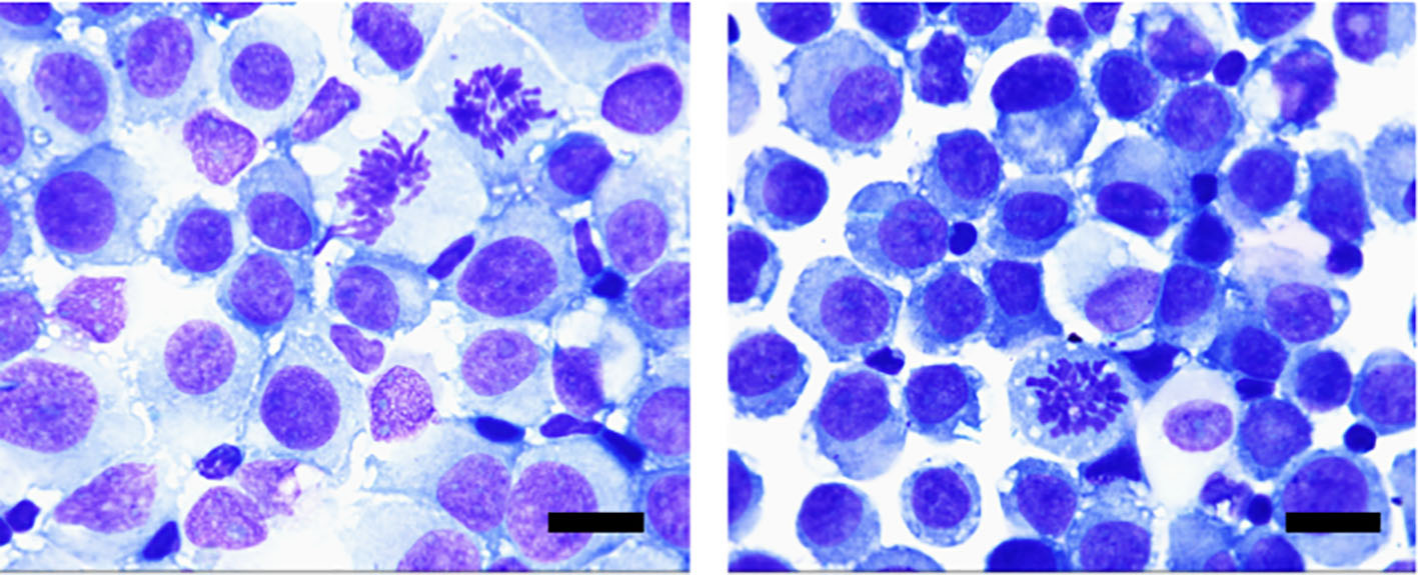
Morphological features of tumor cells of CSF: Cells of different sizes, large cell bodies, and nuclei often deviated to one side, darkened cytoplasm, and rich in vacuoles, mostly blue. Tumor cells in the dividing phase can also be seen. (Wright-Giemsa staining, ×100; scale bar=10μm).

The cytological morphology of the cerebrospinal fluid cells is different from that of the primary tumor as shown in **Figure 4**. The tumor cells often appear singly and scattered, with enlarged and deeply stained nuclei, an increased ratio of nuclear to cytoplasmic volume, and abnormal nuclear divisions and nuclear displacement can also be seen. Some tumor cells have large vacuoles within their cytoplasm. The worst grades of toxicity in each patient during the intrathecal methotrexate therapy are summarized in **Table 3**. Four patients experienced mild bone marrow suppression after intrathecal injection of MTX, five patients developed oral mucosal ulcers, two patients had abnormal liver enzymes, and three patients experienced nausea.

**Table 3:** Toxicity profiles of six patients.

Patient No.	Leukopenia	Neutropenia	Hemoglobin	Thrombocytopenia	AST	ALT	Nausea	Oral mucositis
1	2	2	2	2	0	0	1	2
2	0	0	0	1	0	0	0	0
3	0	0	0	0	1	1	1	1
4	0	0	1	0	0	0	1	1
5	1	1	1	0	2	2	0	1
6	0	0	0	0	0	0	0	1

## Discussion

In this case series, we present the clinical courses and outcomes of 6 patients with leptomeningeal metastasis of gastric cancer (LM-GA) who were treated with intrathecal methotrexate injection. Our findings underscore the potential of this treatment modality in managing a condition that is both rare and challenging. Due to the development of new drugs, including molecular targeted and immunotherapy agents, advancements in systemic therapy have extended the survival of cancer patients beyond what was seen in the 20th century. The 5-year survival rate for patients with advanced gastric cancer treated with systemic therapy is approximately 20-30%. Therefore, it is anticipated that the incidence of leptomeningeal carcinomatosis (LM) complicating gastric cancer will increase with the prolonged survival brought about by advances in systemic therapy. However, because the condition of LM-GC is relatively rare, there are currently no guideline for this disease state. The rate of blood-brain barrier penetration of current systemic chemotherapy regimens is low, while intrathecal injection can bypass the blood-brain barrier, utilizing the cerebrospinal fluid circulation to kill tumor cells with a smaller dose (7). Previous studies have indicated that intrathecal methotrexate (MTX) injection can significantly alleviate the symptoms of patients with leptomeningeal metastasis and improve prognosis. Therefore, this study retrospectively analyzed the local therapeutic effects of six patients with leptomeningeal metastasis from gastric cancer. After the onset of leptomeningeal metastasis, the addition of intrathecal MTX injection treatment significantly improved the patients’ clinical symptoms and quality of life. By incorporating this local chemotherapy method, an overall survival (OS) of 7.5 months was achieved, longer than previously reported for patients with leptomeningeal metastasis from gastric cancer. In addition, the reduction of protein levels in the cerebrospinal fluid also corroborates the benefits brought about by this therapeutic approach.

Notably, this aligns with the innovative approach reported by Jiao et al (8), where a combination of lapatinib, trastuzumab, and capecitabine provided rapid symptomatic relief in a HER2-positive gastric cancer patient with LM-GC. The prevalence of these aggressive histological types in our study mirrors the results of a previous study, further validating the well-established link between these characteristics and advanced gastric cancer stages (9–11). The uniformity of these findings across different patient populations underscores the importance of considering histological features in the management and prognosis of LM-GC.

Our case series findings are consistent with the existing literature, revealing that the majority of patients with LM-GC had advanced disease characterized by Bormann type III or IV (9–11), poorly differentiated, or signet-ring cell histopathology. This histological profile is notably associated with a higher propensity for distant metastasis and a generally poor prognosis, a pattern observed in our patient cohort and corroborated by the multi-center retrospective analysis (12). Furthermore, the case series from Lee et al (13) highlights the aggressive nature and the diagnostic challenges of LM-GC, with MRI proving to be a more sensitive diagnostic tool than CT. For some patients with leptomeningeal metastasis from solid tumors in our center, other lesions of the solid tumors are in a relatively stable state, with only leptomeningeal metastasis showing progression. Moreover, previous studies have suggested that due to the uniqueness of the tumor microenvironment in leptomeningeal metastasis, its genetic characteristics and evolutionary direction are different from those of extracranial lesions.

While our findings corroborate previous research, it is essential to recognize the limitations inherent in our study design. The small sample size, which is a common challenge in studies focusing on rare conditions like LM-GC, may have influenced the generalizability of our results. Future research should aim to expand upon these findings with larger, multicenter studies and consider the other characteristics, such as genomic alterations of CSF cell-free DNA (14), which may provide new insights into the molecular mechanisms of LM-GC and guide targeted therapies.

In conclusion, our case series, along with the existing literature (3, 6), suggests that intrathecal methotrexate injection may offer a viable therapeutic option for patients with LM-GC. The potential for improved patient outcomes, as demonstrated in the case by Jiao et al (8), and the prognostic significance of cytological remission, as indicated by Oh et al (12), highlight the necessity for the development of effective treatment strategies for LM-GC. As the incidence of LM-GC may rise with advancements in systemic chemotherapy, it is imperative that we continue to explore novel therapeutic approaches and biomarkers for early detection and intervention.
